# Pharmacological Inhibition of p38 Mitogen-Activated Protein Kinases Affects KC/CXCL1-Induced Intraluminal Crawling, Transendothelial Migration, and Chemotaxis of Neutrophils *In Vivo*

**DOI:** 10.1155/2013/290565

**Published:** 2013-03-04

**Authors:** Najia Xu, Mokarram Hossain, Lixin Liu

**Affiliations:** Department of Pharmacology, College of Medicine, University of Saskatchewan, 107 Wiggins Road, Saskatoon, SK, Canada S7N 5E5

## Abstract

p38 mitogen-activated protein kinase (MAPK) signalling is critical in the pathophysiology of a variety of inflammatory processes. Leukocyte recruitment to the site of inflammation is a multistep process governed by specific signalling cascades. After adhesion in the lumen, many leukocytes crawl to optimal sites at endothelial junctions and transmigrate to extravascular tissue in a Mac-1-dependent manner. The signalling mechanisms that regulate postadhesion steps of intraluminal crawling, transmigration, and chemotaxis in tissue remain incompletely understood. The present study explored the effect of p38 MAPK inhibitor SB203580 on various parameters of neutrophil recruitment triggered by chemokine KC (CXCL1) gradient. Neutrophil-endothelial interactions in microvasculature of murine cremaster muscle were determined using intravital microscopy and time-lapsed video analysis. SB203580 (100 nM) did not change leukocyte rolling but significantly attenuated neutrophil adhesion, emigration, and transmigration and impaired the initiation of neutrophil crawling and transmigration. In response to KC chemotactic gradient, SB203580 significantly reduced the velocity of migration and chemotaxis index of neutrophils in tissue. The upregulation of Mac-1 expression in neutrophils stimulated by KC was significantly blunted by SB203580 *in vitro*. Collectively, our findings demonstrate that pharmacological suppression of p38 MAPK significantly impairs multiple steps of neutrophil recruitment *in vivo*.

## 1. Introduction

During acute inflammation, leukocytes, mostly neutrophils, are recruited to the afflicted site by a well-defined and dynamic multistep process which includes leukocyte tethering, rolling, adhesion, and transmigration out of the vasculature [1–3]. *In vivo*, neutrophil recruitment is complex and regulated by a variety of molecules and signalling cascades triggered by the cross-talk between neutrophils and endothelium. Neutrophil rolling is mediated by selectins, and adhesion is mediated by *β*2 integrins while emigration is regulated by the interactions between integrins, PECAM-1, and junctional adhesion molecules and their respective ligands [4]. Recently, an additional step of neutrophil recruitment termed intraluminal crawling was described, a process which is molecularly distinct from adhesion [5]. Intraluminal crawling enables neutrophils to reach optimal emigration sites at endothelial junctions independently of hemodynamic forces [6, 7]. The *β*2 integrin LFA-1 mediates firm adhesion of neutrophils to endothelial cells while the subsequent step of intraluminal crawling occurs as a result of interactions between Mac-1 and endothelial ICAM-1 [5]. Blocking of LFA-1 and Mac-1 in monocytes and ICAM-1 and ICAM-2 on endothelial cells was shown to prevent the intraluminal locomotion of monocytes to endothelial cell junctions and ensuing diapedesis [8]. Signalling mechanisms that regulate intraluminal crawling and subsequent transendothelial migration of leukocytes are not completely understood.

p38 mitogen-activated protein kinase (MAPK) signalling regulates a wide array of inflammatory processes, cell proliferation, differentiation, survival, and invasion [9, 10]. p38 MAPK signalling participates in the expression and function of inflammatory cytokines such as TNF*α*, IL-1, IL-2, IL-6, IL-7, and IL-8 [11]. Pharmacological inhibition of p38 MAPK signalling was previously shown to ameliorate inflammatory disorders such as asthma, rheumatoid arthritis, inflammatory bowel disease, stroke, systemic lupus erythematosus, and autoimmune diseases [12–17], thus serving as a promising therapeutic target [18]. Previous studies have documented the role of p38 MAPK in neutrophil function during different steps of neutrophil recruitment. Cytokines and inflammatory mediators such as TNF*α*, LPS and fMLP, and chemokine KC/CXCL1 were shown to phosphorylate p38 MAPK during inflammation [19–21]. Pharmacological inhibition of p38 MAPK was found to impair neutrophil chemotaxis and transendothelial migration induced by the chemokine KC [21]. In this latter study, the number of emigrated neutrophils in each grid of defined distance from the observed venule was measured as neutrophil chemotaxis parameter. However, the dynamic motile behavioural changes of chemotaxing neutrophils such as velocity of migration and chemotaxis index were not measured, and the role of p38 MAPK in these changes was not determined. In addition, whether there is a role of p38 MAPK in intraluminal crawling of neutrophils has not been reported. 

SB203580 is a widely used selective inhibitor of the p38*α* and p38*β* isoforms and binds to the active site of p38 MAPK in an ATP-competitive manner [22]. SB203580 can further block the translocation of p38 MAPK and its downstream substrate MAPK activated protein kinase 2 from the nucleus to cytosol [23]. Mounting evidence suggests that SB203580 is effective in a variety of *in vitro* and *in vivo* models of inflammation characterized by attenuated production of proinflammatory cytokines such as TNF*α* and IL-1*β* [12]. 

The use of real-time intravital microscopy and time-lapsed video photography analysis makes it possible to determine simultaneously multiple leukocyte recruitment parameters of rolling, adhesion, emigration, intraluminal crawling velocity, transmigration time, detachment time, migration velocity, chemotaxis velocity, and chemotaxis index in tissue [5, 24]. In the present study, we explored the effects of the p38 MAPK inhibitor SB203580 on various parameters of neutrophil recruitment induced by chemotactic gradient of KC *in vivo*. 

## 2. Materials and Methods

### 2.1. Mice

C57BL/6 mice were purchased from Charles River Canada (Saint-Constant, QC, Canada). Male mice between 8- and 16-week-old were used in experiments. This study was carried out with the approval of animal protocols from the University Committee on Animal Care and Supply (UCACS) at the University of Saskatchewan following the standards of the Canadian Association of Animal Care.

### 2.2. Measurement of Neutrophil Recruitment by Intravital Microscopy

Mice were anesthetised using an i.p. injection of 10 mg/kg xylazine (Bayer, Toronto, ON, Canada) and 200 mg/kg ketamine hydrochloride (Rogar, Montreal, QC, Canada). The mouse cremaster muscle preparation was used to study neutrophil behaviour in microcirculation and tissue as described previously [24–26]. The cremaster muscle was superfused with 37°C warmed bicarbonate-buffered saline (pH 7.4; containing in mM 133.9 NaCl, 4.7 KCl, 1.2 MgSO_4_, and 20 NaHCO_3_). An upright microscope (model Eclipse Ci-s, Nikon) with a LUCPLFLN 20x objective lens was connected to a CCD color video camera (DC-220, Dage) for bright-field intravital microscopy. For the induction of neutrophil recruitment, an agarose gel at 1 mm^3^ size containing the optimal concentration of CXC chemokine KC/CXCL1 (0.5 *μ*M; Peprotech, Dollard des Ormeaux, QC, Canada) was placed on the surface of the cremaster muscle in a preselected area 350 *μ*m from and parallel to the observed postcapillary venule. After placing a coverslip, the cremaster muscle was superfused with bicarbonate-buffered saline at a very slow rate (≤10 *μ*L/min) for allowing formation of chemoattractant gradient. Throughout the experiment, neutrophil behaviour and hemodynamic changes in the selected cremasteric postcapillary venule (25−40 *μ*m diameter) were visualized, projected on a TV monitor, recorded at real time on a DVD recorder before (for time 0 min) and after addition of KC-gel (recorded for 60 or 90 min). During recording, all efforts were made to adjust and keep the microscope focused on the adhering, crawling, transmigrating, and chemotaxing neutrophil inside the venule and in the muscle tissue. The number of rolling, adherent, and emigrated neutrophils during offline playback analysis of the recorded video was determined in the cremasteric microvasculature as described previously [24–26].

### 2.3. Inhibition of p38 MAPK

 The inhibitor SB203580 (EMD, Billenca, MA) was used to experimentally suppress the activity of p38 MAPK. SB203580 was dissolved in DMSO at 10 mM and stored in aliquots at −20°C. To determine the role of p38 MAPK in neutrophil crawling and transmigration, SB203580 (100 nM) was superfused 30 min prior to the placement of KC-containing gel and SB203580 superfusion remained for the duration of KC treatment (60 min). To analyze the role of p38 MAPK in neutrophil migration and chemotaxis in cremasteric tissue after transendothelial migration, SB203580 was superfused 30 min after the placement of KC-containing gel when at least 8 emigrated neutrophils were identified in perivascular tissue and remained perfused for additional 60 min. SB203580 administered intravenously before this KC treatment completely inhibited adhesion and emigration induced by KC (data not shown). For *in vitro* experiments, SB203580 (10 *μ*M) was added to the medium 30 min prior to stimulation of neutrophils with KC. In all experiments, the same amount of DMSO as in the SB203580-treated group was used in the control group without SB203580 and was not found to affect any responses. 

### 2.4. Cell Tracking and Analysis of Leukocyte Recruitment Parameters

 Using ImageJ software, leukocyte intraluminal crawling, transmigration, and chemotaxis in cremasteric vasculature were analysed using the time-lapsed movie converted from the real-time video recording of the experiment as described previously [5, 7, 24]. The following recruitment parameters were quantified from tracking and analyzing at least 40 cells for each treatment group.

- Percentage of adherent leukocytes that crawled: the number of adherent and then crawled leukocytes divided by the total number of adherent leukocytes (%).
- Crawling distance: the total distance the leukocyte crawled from the initial site of adhesion to the transmigration site (*μ*m).
- Crawling time: duration of leukocytes undergoing intraluminal crawling (min).
- Velocity of crawling: the crawling distance in the vessel lumen divided by crawling time (*μ*m/min).
- Percentage of crawling leukocytes along the blood flow: the number of crawling leukocytes with their final crawling location in the direction of blood flow divided by the total crawling leukocytes (%).
- Directional crawling index: the ratio of the crawling distance in the direction toward the KC gel to the total crawling distance that the cell crawled in the lumen of the venule.
- Percentage of crawling leukocytes that transmigrated: the number of crawled and then transmigrated leukocytes divided by the total number of crawling leukocytes (%).
- Transmigration time: from the time the leukocyte stopped crawling and started to transmigrate across endothelium to the time the whole leukocyte body was just outside the venule (min).
- Detachment time: from the time the leukocyte body was just outside the venule after its transmigration to the time when the leukocyte migrated away and lost contact to the venule (min).
- Migration distance: the sum of the distance that the leukocyte moved from the start point after detachment from the venule to the end point of the migration in tissue at 60 or 90 min of KC treatment or to the end point of the field of view (*μ*m).
- Velocity of migration: migration distance in tissue divided by migration time (*μ*m/min).
- Chemotaxis index in tissue: the ratio of total chemotaxis distance, that is, the distance in the direction toward the KC-gel, to the total migration distance that the cell moved in tissue.

### 2.5. Isolation of Murine Neutrophils

 Femurs and tibias from mice were isolated and the marrow flushed with ice-cold Ca^2+^-free and Mg^2+^-free phosphate-buffered saline (PBS) solution. Neutrophils were isolated using a Percoll (GE Healthcare, Uppsala, Sweden) gradient (72%, 64%, and 52%) centrifugation at 1060 g for 30 min as described previously [27] and subsequently were washed with PBS.

### 2.6. FACS Analysis of Neutrophil Mac-1 and LFA-1 Expression

The expression of Mac-1 was determined using a previously described method with slight modifications [28]. Following lysis of red blood cells, bone-marrow-derived neutrophils were incubated at 37°C for 30 min in the presence or absence of 10 *μ*M SB203580 *in vitro*. The cells were stimulated with KC (5 nM for 10 min) to upregulate Mac-1 or LFA-1 expression. Aliquots of the neutrophil suspension (10^6^/mL) were washed in ice-cold PBS containing 0.5% BSA, stained with a fluorescent anti-Mac-1 antibody (Anti-mouse CD11b FITC; clone M1/70; eBioscience, San Diego, CA) or its respective isotype control (Rat IgG2b*κ* FITC; eBioscience) or fluorescent anti-LFA-1 antibody (Anti-mouse CD11a FITC; clone M17/4; eBioscience) or its respective isotype control (Rat IgG2a*κ* FITC; eBioscience), and incubated for 30 min at 4°C. The samples were then centrifuged (1200 rpm, 3 min, 4°C) and washed twice with ice-cold PBS containing 0.5% BSA and were analysed in the FL-1 channel of an Epics XL flow cytometer (Beckman Coulter, Miami, FL) with an excitation wavelength of 488 nm and an emission wavelength of 530 nm. 

### 2.7. Statistical Analysis

Data are expressed as means ± SEM. *n* denotes the number of mice studied in each group or the number of mice used to derive bone marrow neutrophils for* in vitro *studies. Statistical analysis was made using Student's *t*-test, and values of *P* < 0.05 were considered statistically significant.

## 3. Results

Previous studies have shown and we have confirmed in our model system that using neutrophil-selective chemoattractants such as CXC chemokine KC or MIP-2, more than 95% of the adherent or emigrated leukocytes were found to be neutrophils [5, 21, 26]. Therefore, in this study we use terms of leukocytes and neutrophils interchangeably when dealing with the cell type in different recruitment parameters. 

**Figure 1:**
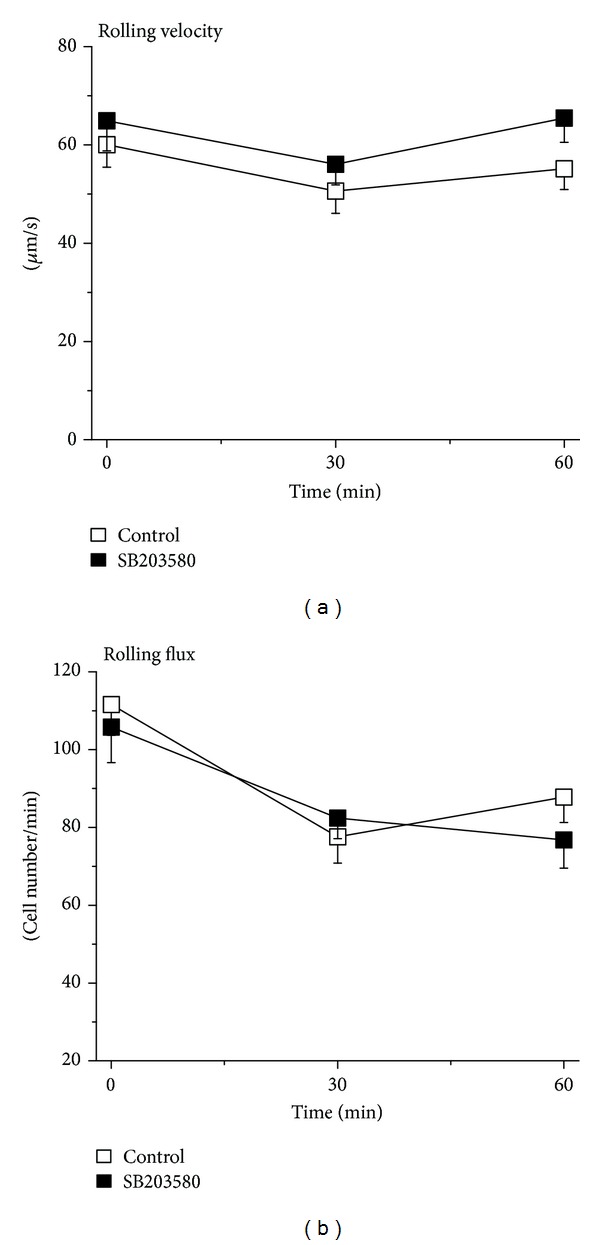
Effect of SB203580 on KC-induced neutrophil rolling. (a) Time course of the velocity of neutrophil rolling (*μ*m/sec) triggered by KC in the absence (control, white) and in the presence (black) of p38 MAPK inhibitor SB203580 (100 nM) 30 min prior to and 60 min following the placement of KC-containing gel. Data are means ± SEM (*n* = 5). (b) Time course of the flux of rolling neutrophils (cells/min) triggered by KC in the absence (control, white) and in the presence (black) of p38 MAPK inhibitor SB203580 (100 nM) 30 min prior to and 60 min following the placement of KC-containing gel. Data are means ± SEM (*n* = 5).

As a first step, the effect of SB203580 on the early neutrophil recruitment process of rolling was analyzed using intravital microscopy. To this end, superfusion of murine cremaster muscles with SB203580 (100 nM) for 30 min prior to and for 1 h following the placement of KC gel did not significantly modify the rolling flux and rolling velocity of neutrophils as compared to the control saline-superfused cremaster muscle (*P* > 0.05; Figures 1(a) and 1(b)). 

**Figure 2:**
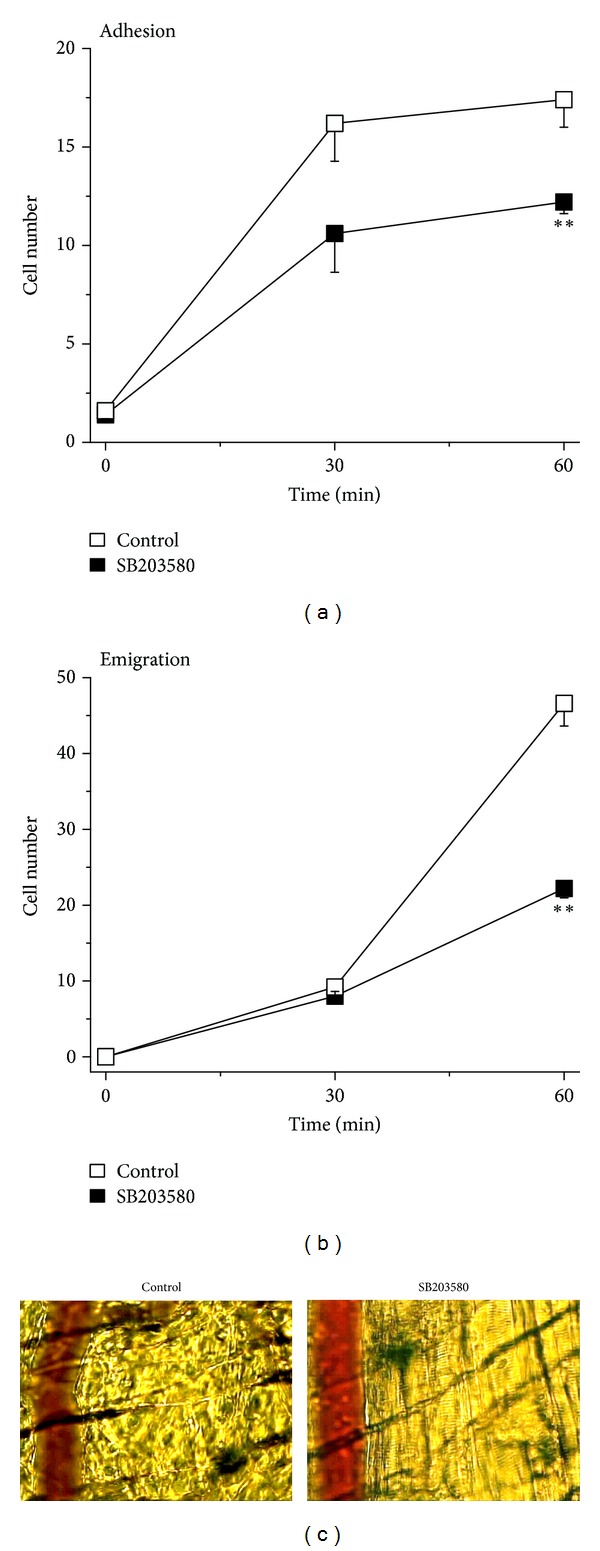
Effect of SB203580 on KC-induced neutrophil adhesion and emigration. (a) Time course of the number of adherent neutrophils (cells/100 *μ*m venule) triggered by KC in the absence (control, white) and in the presence (black) of p38 MAPK inhibitor SB203580 (100 nM) 30 min prior to and 60 min following the placement of KC-containing gel. Data are means ± SEM (*n* = 5). ∗∗ indicates significant difference (*P* < 0.01) from the control without SB203580 (*t*-test). (b) Time course of the number of emigrated neutrophils (cells/235 × 208 *μ*m^2^ field) triggered by KC in the absence (control, white) and in the presence (black) of p38 MAPK inhibitor SB203580 (100 nM) 30 min prior to and 60 min following the placement of KC-containing gel. Data are means ± SEM (*n* = 5). ∗∗ indicates significant difference (*P* < 0.01) from the control without SB203580 (*t*-test). (c) Representative images from intravital video microscopy showing a postcapillary venule and the surrounding cremasteric tissue with adherent and emigrated neutrophils triggered by KC for 60 min and in the absence (left panel, control) and in the presence of SB203580 (right panel, SB203580).

To analyse the subsequent steps of neutrophil recruitment in the same cremaster muscles, we further quantitatively visualized and determined the number of adherent and emigrated neutrophils. As depicted in Figures 2(a) and 2(c), the number of adherent neutrophils had a tendency of reduction at 30 min and decreased significantly at 60 min after stimulation with KC in SB203580-superfused cremaster muscle as compared to saline superfusion. Similarly, the number of emigrated neutrophil was not reduced until 60 min KC treatment in SB203580-treated cremaster muscles as compared to the saline-superfusion control (Figures 2(b) and 2(c)). Interestingly, the same treatment with SB203580 reduced leukocyte adhesion by 32% and leukocyte emigration by 50% at 60 min after KC treatment. This suggests that there is a different effect of SB203580 on leukocyte adhesion and emigration and there may be additional inhibition by the inhibitor on other recruitment steps such as intraluminal crawling and transendothelial migration that caused more substantial suppression in emigration.

**Figure 3:**
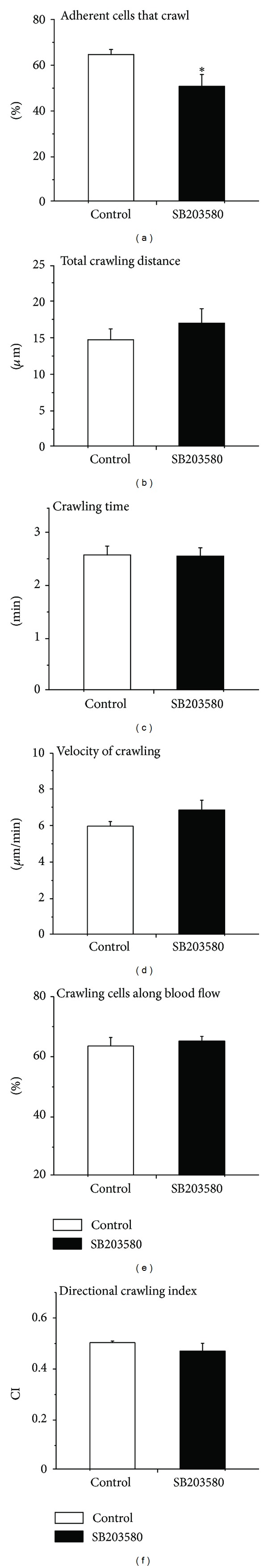
Effect of SB203580 on parameters of KC-induced intraluminal crawling of neutrophils. (a) The percentage of adherent cells that crawl in the luminal surface of the endothelium upon stimulation with KC in the absence (control, white) and in the presence (black) of p38 MAPK inhibitor SB203580 (100 nM) 30 min prior to and 60 min following the placement of KC-containing gel. Data are means ± SEM (*n* = 5). ∗ indicates significant difference (*P* < 0.05) from the control without SB203580 (*t*-test). (b) The total distance (*μ*m) traversed by a crawling neutrophil (averaged from >40 cells) in the luminal surface of the endothelium upon stimulation with KC in the absence (Control, white) and in the presence (black) of p38 MAPK inhibitor SB203580 (100 nM) 30 min prior to and 60 min following the placement of KC-containing gel. Data are means ± SEM (*n* = 5). (c) The duration (min) of neutrophil crawling in the luminal surface of the endothelium upon stimulation with KC in the absence (control, white) and in the presence (black) of p38 MAPK inhibitor SB203580 (100 nM) 30 min prior to and 60 min following the placement of KC-containing gel. Data are means ± SEM (*n* = 5). (d) The velocity (*μ*m/min) of neutrophil crawling in the luminal surface of the endothelium upon stimulation with KC in the absence (control, white) and in the presence (black) of p38 MAPK inhibitor SB203580 (100 nM) 30 min prior to and 60 min following the placement of KC-containing gel. Data are means ± SEM (*n* = 5). (e) The percentage of crawling neutrophils that crawl along the blood flow in the luminal surface of the endothelium upon stimulation with KC in the absence (control, white) and in the presence (black) of p38 MAPK inhibitor SB203580 (100 nM) 30 min prior to and 60 min following the placement of KC-containing gel. Data are means ± SEM (*n* = 5). (f) The directional crawling index (CI) of neutrophil in the luminal surface of the endothelium upon stimulation with KC in the absence (control, white) and in the presence (black) of p38 MAPK inhibitor SB203580 (100 nM) 30 min prior to and 60 min following the placement of KC-containing gel. Data are means ± SEM (*n* = 5).

 Next, we assessed the effects of pharmacologically inhibiting p38 MAPK on neutrophil intraluminal crawling and transendothelial migration. To address this, the intermediate step between adhesion and emigration, the intraluminal crawling that follows KC-triggered adhesion, was studied using time-lapsed video microscopy. As shown in Figure 3(a), the percentage of adherent neutrophils that proceeded to intraluminal crawling was significantly reduced upon treatment with SB203580. Interestingly, treatment with SB203580 did not significantly modify the total distance traversed by crawling neutrophils and the time taken (Figures 3(b) and 3(c)). Accordingly, the velocity of crawling was not significantly altered upon treatment with SB203580 (Figure 3(d)). In addition, neither the directionality of intraluminal crawling relative to blood flow nor the directional crawling index was significantly different in the two groups (Figures 3(e) and 3(f)).

**Figure 4:**
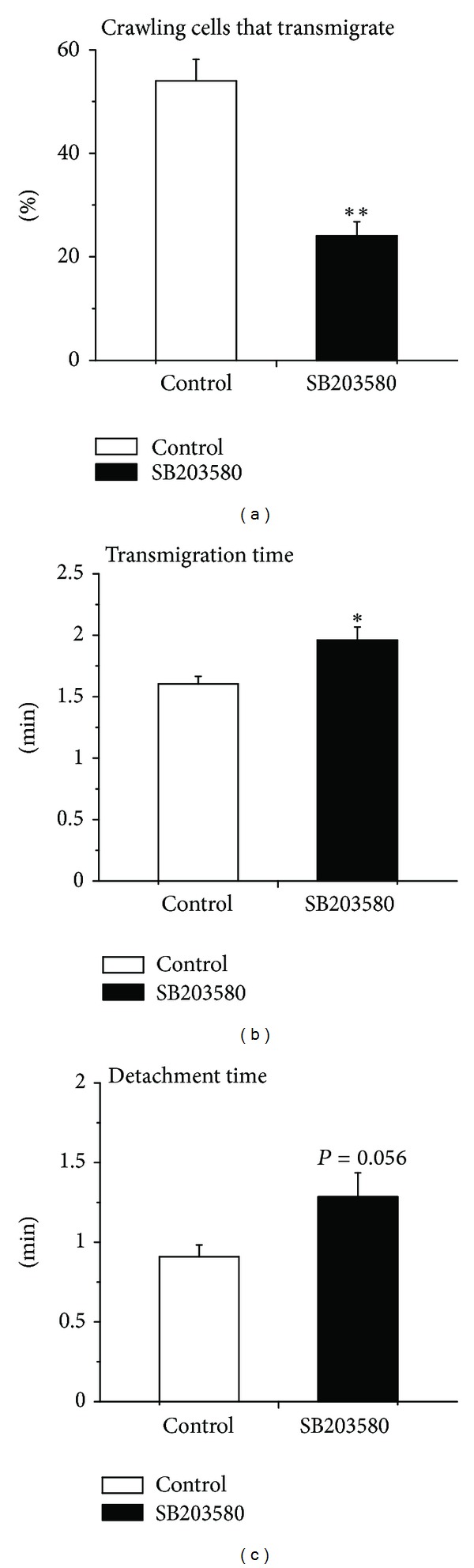
Effect of SB203580 on parameters of KC-induced neutrophil transmigration. (a) The percentage of crawling cells that transmigrate across the endothelium upon stimulation with KC in the absence (control, white) and in the presence (black) of p38 MAPK inhibitor SB203580 (100 nM) 30 min prior to and 60 min following the placement of KC-containing gel. Data are means ± SEM (*n* = 5). ∗∗ indicates significant difference (*P* < 0.01) from the control without SB203580 (*t*-test). (b) The duration (min) of neutrophil transmigration across the endothelium upon stimulation with KC in the absence (control, white) and in the presence (black) of p38 MAPK inhibitor SB203580 (100 nM) 30 min prior to and 60 min following the placement of KC-containing gel. Data are means ± SEM (*n* = 5). ∗ indicates significant difference (*P* < 0.05) from the control without SB203580 (*t*-test). (c) Detachment time (min) of transmigrated neutrophils from the venule upon stimulation with KC in the absence (control, white) and in the presence (black) of p38 MAPK inhibitor SB203580 (100 nM) 30 min prior to and 60 min following the placement of KC-containing gel. Data are means ± SEM (*n* = 5). The statistical difference between the two groups was found to be *P* = 0.056 (not significant; *t*-test).

 A decrease in the number of emigrated neutrophils could have resulted from impaired transendothelial migration following intraluminal crawling. To test this possibility, we further determined various parameters of transendothelial migration. Clearly, the percentage of crawling neutrophils that proceeded to transendothelial migration was significantly lower in SB203580-superfused than in only saline-superfused control cremaster muscle (Figure 4(a)). Determination of transmigration time revealed that neutrophils in SB203580-superfused cremaster muscle required significantly longer time as compared to the control group reflecting impaired migration of neutrophils across the endothelial layer (Figure 4(b)). We further analysed the time required for detachment of neutrophils from the endothelial cells prior to their chemotaxis. As illustrated in Figure 4(c), the detachment time in SB203580-superfused cremaster muscle tended to be higher than the control, an effect not reaching statistical significance. 

**Figure 5:**
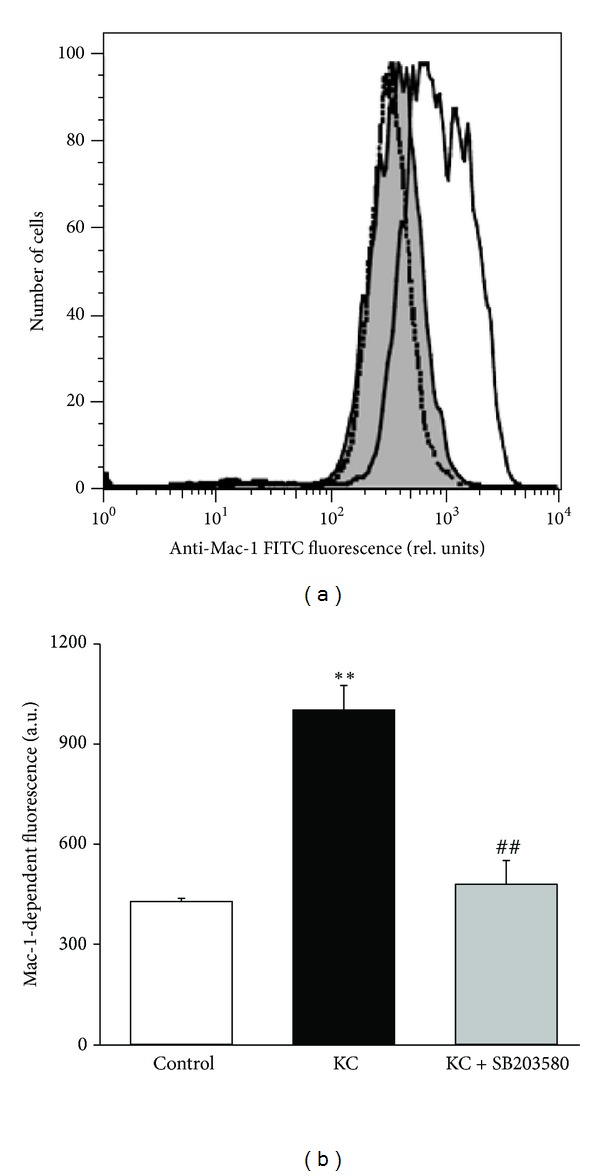
Effect of SB203580 on neutrophil Mac-1 expression *in vitro*. (a) Original histogram of Mac-1-dependent fluorescence in bone-marrow-derived neutrophils in the absence (gray shadow) or in the presence of stimulation with KC (5 nM for 10 min; black line). Neutrophils treated with p38 MAPK inhibitor SB203580 (10 *μ*M for 40 min) are shown as a dotted line. (b) Means ± SEM (*n* = 3) of Mac-1-dependent fluorescence expressed as geomeans in unstimulated neutrophils (control; white bar) and in neutrophils stimulated with KC (black bar) and with KC plus SB203580 (gray bar). ∗∗ indicates significant difference (*P* < 0.01) from the control without KC stimulation and ## indicates significant difference (*P* < 0.01) from KC stimulation without SB203580 (*t*-test).

 As Mac-1 expression on neutrophil surface is important for the intraluminal crawling and subsequent transendothelial migration of neutrophils, we further analysed whether or not SB203580 modulated the expression of Mac-1 *in vitro*. To this end, stimulation with KC resulted in significantly increased expression of Mac-1 on neutrophils. Treatment with SB203580 (10 *μ*M) virtually abrogated KC-triggered upregulation of Mac-1 expression (Figures 5(a) and 5(b)). We also found that the treatment of neutrophils with KC *in vitro* did not change the expression level of LFA-1, and SB203580 (10 *μ*M) did not affect the expression level of LFA-1 after stimulation with KC (data not shown).

The reduced intraluminal crawling and transendothelial migration upon SB203580 treatment would subsequently result in a decrease in the migration and chemotaxis of emigrated neutrophils in cremasteric tissue. To have some chemotaxing neutrophils in the tissue, SB203580 was, therefore, superfused on the cremaster muscle after their transmigration into the tissue; that is, superfusion of cremaster muscle with SB203580 was started at 30 min after KC-gel addition when about 8 or more leukocytes were identified being just emigrated in perivascular tissue, and SB203580 remained perfused for 60 min in the presence of KC-gel. Stimulation of KC enhanced rolling velocity, rolling flux, and the number of adherent and emigrated neutrophils. Neutrophil rolling velocity and rolling flux at 90 min following KC treatment (64.0 ± 4.8 *μ*m/sec and 92.6 ± 4.6 cells/min, resp.; *n* = 3) were not significantly modified by the presence of 100 nM SB203580, added at 30 min after KC stimulation (55.3.0 ± 6.5 *μ*m/sec and 76.6 ± 10.9 cells/min, resp.; *n* = 3). Similarly, neutrophils adhesion and emigration at 90 min following KC treatment (12.3 ± 0.1 cells/100-*μ*m venule and 57.6 ± 0.7  cells/235 × 208 *μ*m^2^ field, resp.; *n* = 3) were not significantly modified by superfusion with 100 nM SB203580 started at 30 min following KC stimulation (10.3 ± 0.8 *μ*m/sec and 55.0 ± 5.0 cells/min, resp.; *n* = 3). 

**Figure 6:**
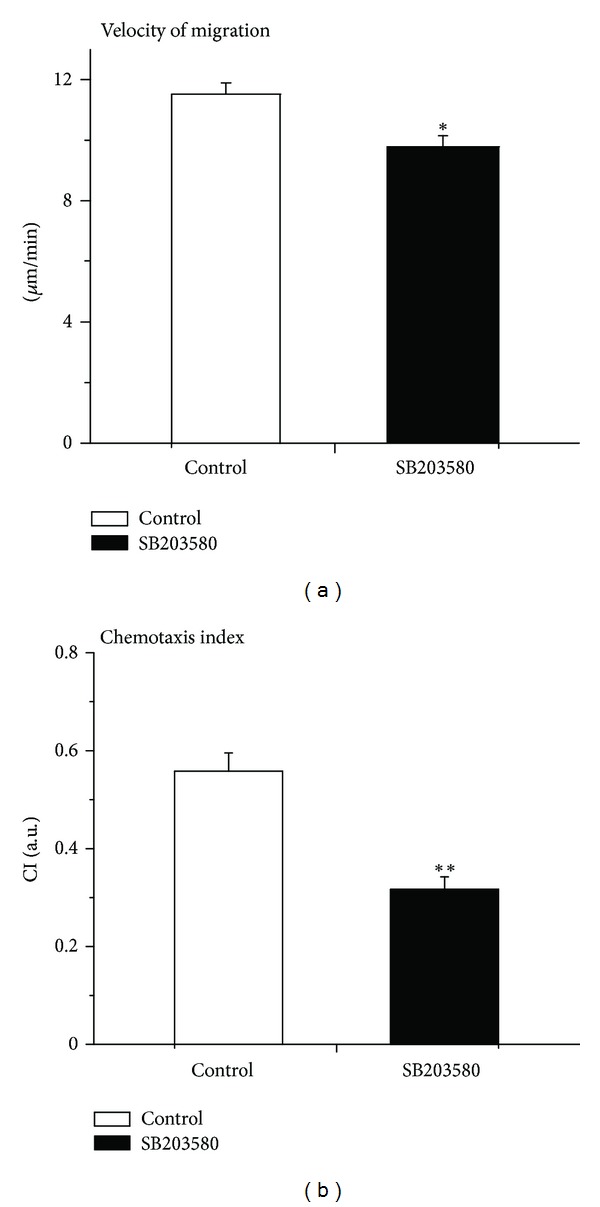
Effect of SB203580 on KC-induced neutrophil migration and chemotaxis. (a) The velocity of neutrophil migration in response to KC stimulation in the absence (control, white) and in the presence (black) of p38 MAPK inhibitor SB203580 (100 nM) 30 min following the placement of KC-containing gel. Data are means ± SEM (*n* = 5). ∗ indicates significant difference (*P* < 0.05) from the control without SB203580 (*t*-test). (b) The chemotaxis index (CI) in response to KC stimulation in the absence (control, white) and in the presence (black) of p38 MAPK inhibitor SB203580 (100 nM) 30 min following the placement of KC-containing gel. Data are means ± SEM (*n* = 5). ∗∗ indicates significant difference (*P* < 0.01) from the control without SB203580 (*t*-test).

After observing no significant changes in leukocyte rolling, adhesion, and emigration with SB203580 after KC treatment, we then employed time-lapsed video analysis to determine the parameters of neutrophil migration and chemotaxis towards chemoattractant KC gel in cremasteric tissue. Administration of SB203580 modestly but significantly attenuated the velocity of neutrophil migration as compared to saline control (Figure 6(a)). Similarly, as illustrated in Figure 6(b), neutrophil chemotaxis index was significantly decreased in SB203580-superfused cremasteric tissue in mice when compared with chemotaxis index in saline-superfused control mice. This suggests that pharmacological inhibition of p38 MAPK by SB203580 reduced neutrophil migration and more substantially neutrophil chemotaxis in extravascular tissue. 

## 4. Discussion

In this study, we demonstrate the effects of the p38 MAPK inhibitor SB203580 on the modulation of different steps of neutrophil recruitment. We found that *in vivo* treatment with SB203580 does not appreciably alter the parameters of neutrophil intraluminal crawling but significantly modifies cellular functions during transendothelial migration and chemotaxis in tissue. Our results reveal an important role of p38 MAPK in various steps of neutrophil recruitment particularly the initiation of intraluminal crawling and transmigration, transmigration time, and migration and chemotaxis in tissue. 

Acquiring the knowledge of the functional role of p38 MAPK in leukocyte recruitment is in large part dependent on the pharmacological inhibition of the kinase by SB203580 [21, 29–33]. However, more recent studies highlight that the limited specificity of SB203580 precludes safe conclusions in regards to the precise role of p38 MAPK in the regulation of leukocyte recruitment [34, 35]. Earlier studies documented that SB203580 does not inhibit other kinases of the MAPK family at relatively high molar concentrations (≤100 *μ*M) [36]. It is obvious that due to the lack of cellular specificity *in vivo*, the inhibition of endothelial p38 MAPK by SB203580 treatment may not be ruled out. To the best of our knowledge, this is the first study that systematically analysed the effect of SB203580 on each step of neutrophil recruitment cascade *in vivo*.

Administration of SB203580 prior to the stimulation with KC resulted in significantly lower adhesion and emigration of neutrophils. However, administration of SB203580 30 min following stimulation with KC dissipated the differences in adhesion and emigration. Our emigration data are in agreement with previous reports where both p38 MAPK inhibitors SB203580 and SKF86002 significantly reduced the number of emigrated cells [21]. We observed a robust decrease in the percentage of crawling neutrophils that transmigrated in response to KC as the result of SB203580 treatment. In contrast to the previous report [21], we observed a slight but significant reduction in the number of neutrophils adhering to the microvasculature. In view of this discrepancy, previous studies support our findings that p38 MAPK plays a role in neutrophil adhesion [37–40], an effect that is in part mediated by downregulation of *β*2 integrin expression [41]. 

 The transition from adhesion to emigration is effectively accomplished by intraluminal crawling of neutrophils from the initial site of adhesion to the junctional extravasation site in endothelium. Although the efficiency of adherent leukocytes to crawl was slightly but significantly diminished in the presence of SB203580, the crawling directionality along the blood flow, the total crawling distance, time, and velocity were not apparently affected. Signalling mechanisms that regulate intraluminal crawling remain elusive. A recent report revealed that Vav1, a guanine exchange factor for the Rho family GTPases Rac and Cdc42, is a key regulator of actin cytoskeletal organization during intraluminal crawling [7]. The mammalian-actin binding protein 1 (mAbp1) was also shown to participate in intraluminal crawling of neutrophils under conditions of physiological shear stress [42]. Both Vav1 and mAbp1 are downstream molecules of the spleen tyrosine kinase (Syk) that plays a crucial role in *β*2 integrin-mediated signalling [42, 43]. By the same token, Syk-mediated signalling was shown to be essential for p38 MAPK activation [44, 45]. It is possible that KC activates p38 MAPK through Syk and regulates intraluminal crawling of neutrophils in our model. 

The chemokine KC elicits Mac-1 expression which mediates intraluminal crawling [5, 46]. We observed that upregulation of Mac-1 expression after stimulation with KC was significantly abated in the presence of SB203580. SB203580 treatment may also impair the transition of adherent leukocytes to the crawling phase without modulating the dynamics of neutrophil crawling *per se*. Interestingly, Mac-1 was previously shown to be downregulated in TNF*α*-stimulated and fMLP-stimulated neutrophils treated with SB203580 [47, 48]. Similarly, the upregulation of Mac-1 expression on neutrophils elicited by the bacterial M1 proteins *in vivo* was significantly abated by the p38 MAPK inhibitors SB239063 and SKF86002 [49]. However, the authors showed that both SB239063 and SKF86002 did not significantly alter Mac-1 expression *in vitro* after stimulation with bacterial M1 protein [49]. It is tempting to speculate that ramifications of p38 MAPK inhibition *in vivo* include dysregulated expression of endothelial junctional adhesion molecules and neutrophil integrins that may contribute to the marked decrease in neutrophil transmigration.

 Previous studies have highlighted the role of p38 MAPK in neutrophil chemotaxis [19, 21]. Cara and coworkers utilized a grid model to quantify the number of chemotaxing neutrophils in several partitions between the vessel and chemoattractant-containing gel. The authors reported a significant reduction in the number of chemotaxing neutrophils in partitions closer to the chemoattractant-containing gel. In line with their findings, our data reveal that inhibition of p38 MAPK decreases the chemotaxis index and velocity of migration which may contribute to the overall impairment of neutrophil chemotaxis by SB203580 as reported previously [21].

 Taken together, our results suggest that the pharmacological p38 MAPK inhibitor SB203580 affects neutrophil recruitment induced by the CXC chemokine KC by modulating various important neutrophil recruitment steps such as neutrophil transendothelial migration and chemotaxis in tissue. Although SB203580 significantly reduced the number of adherent neutrophils that proceeded to crawling, the various functional parameters of intraluminal crawling were not substantially altered, an effect that may be attributed to the modulation of *β*2 integrin Mac-1 expression on neutrophils.
